# The prevalence of household second-hand smoke exposure and its correlated factors in six counties of China

**DOI:** 10.1136/tc.2008.024836

**Published:** 2009-01-08

**Authors:** C-P Wang, S J Ma, X F Xu, J-F Wang, C Z Mei, G-H Yang

**Affiliations:** 1 Weifang Medical College, Shandong, China; 2 Institute of Basic Medical Sciences, Chinese Academy of Medical Sciences, Beijing, China; 3 Chinese Center for Disease Control and Prevention, Beijing, China

## Abstract

**Objective:**

To study the prevalence of, and discuss factors contributing to, household second-hand smoke exposure in six counties in China, providing scientific support for the need to establish tobacco control measures in these areas.

**Methods:**

A cross-sectional survey was performed. Investigators conducted face-to-face interviews using a standardised questionnaire to collect information on demographics, passive smoking behaviours and knowledge, and attitudes towards tobacco control. The setting was six counties from the three provinces: Mianzhu and Xichong counties in Sichuan Province; Anyi and Hukou counties in Jiangxi Province; and Xinan and Yanshi counties in Henan Province. A total of 8142 non-smokers (aged 18–69) in 2004 were included in the data analysis. Household second-hand smoke exposure rate as defined as the proportion of household passive smokers in the non-smoker population was used as the measure of household second-hand smoke exposure.

**Results:**

The analysis of 8142 non-smokers revealed that, in these selected counties, the household second-hand smoke exposure rate was 48.3%. Respondents had positive attitudes towards tobacco control. Of 6972 respondents, 84.4% supported all the three tobacco control policies (banning smoking in public places, banning the selling of cigarettes to minors, banning all cigarette advertisements). In 3165 families with smokers, 87.2% of respondents reported that smokers would smoke in front of them. In 2124 families with smokers and children, 76.5% of respondents reported that smokers would smoke in front of children. As many as 42.1% of non-smokers would offer cigarettes to their guests, and only 46.8% of respondents would ask smokers to smoke outdoors. Only 6.3% of families completely forbade smoking at home. Multivariate logistic regression analysis revealed high second-hand smoke exposure for the following demographic groups: Jiangxi Province inhabitants, females, those with low education level, farmers and married respondents.

**Conclusions:**

Household second-hand smoke exposure rates in the selected counties were high. A high percentage of respondents reported that smokers would smoke in front of them and children. The pressure from non-smokers against smoking was relatively low, although offering cigarette was prevalent. Households that were completely smoking-free were rare, Further studies on these correlated factors could help us establish effective measures to reduce household second-hand smoke exposure.

Second-hand smoke (SHS) exposure, also known as “involuntary smoking” or “passive smoking”, refers to non-smokers’ inhalation of smoke from the exhalation of smokers or burning cigarettes. SHS contains hundreds of toxic and carcinogenic substances, including formaldehyde, benzene, chloroethylene, arsenic, ammonia and hydrocyanic acid. It is aetiologically related to many diseases, including cancer, cardiovascular diseases and many serious respiratory diseases.^1^ ^2^

With the accumulation of scientific evidence about the harm of SHS, many countries have taken action to reduce SHS exposure through legislation and health education, and have achieved significant improvements. In developed countries such as the US and New Zealand, SHS exposure is declining.^3^ ^4^ In China, data on national smoking behaviour in 1996 and 2002 revealed no reduction in the prevalence of SHS exposure over this period, with the reported SHS exposure rate at 53.5% and 51.9% respectively.^5^ ^6^ According to some studies, as many as 540 million Chinese people are exposed to SHS, including 180 million children under the age of 15.^7^ The national survey in 2002 found that exposure rates in urban and rural areas were 49.7% and 54.0% respectively, revealing a higher SHS exposure in rural areas than in urban areas.^5^ Among all possible locations for SHS exposure, the household is the most important.^5^ County residents constitute 76.5% of the Chinese population, according to the national census in 2000.^8^ Thus, reducing household SHS exposure in county areas should be a priority for China’s tobacco control. The study reported in this paper describes and discusses the prevalence of household SHS exposure and its correlated factors in six counties of China, thereby providing a scientific basis for establishing effective interventions to reduce household SHS exposure in counties of China.

## METHODS

### Setting

This study was based on the Fogarty International Center (FIC) of the US National Institues of Health (NIH)-initiated “International Tobacco and Health Research Capacity Building Program”.^9^ This FIC project in China aimed to reduce SHS exposure in county areas. Six counties in three provinces were selected: Mianzhu and Xichong from Sichuan Province, Anyi and Hukou from Jiangxi Province and Xinan and Yanshi from Henan Province. Sichuan province is in southwest China, Jiangxi province is in the southeast and Henan province in the middle of China.

### Sampling

In each county, subjects were selected based on a stratified three-stage random sampling method. In the first stage, we randomly selected 10 villages from rural areas and 10 neighbourhood committees from urban areas from each county, using a computer-generated simple random sampling method. In the second stage, 120 households were randomly selected from the selected village or neighbourhood committee, using the same sampling method. In the third stage, the person (aged 18–69) whose birth date was closest to our survey date was selected as our study subject. In total, 14 400 subjects were sampled.

### Data collection and management

Using a standardised questionnaire, we completed 12 036 face-to-face interviews in 14 400 sampled subjects. The response rate was 83.6% (12 306/14 400). All the data entry was completed independently by two individuals from a professional data entry company to ensure data quality. After data cleaning and logical verification, the final database for analysis was determined. In all, 11 985 questionnaires were validated after data clearance, of which 8142 were non-smokers.

### Measures

Our subjects, 8142 non-smokers, were asked questions about their demographic characteristics, SHS exposure behaviours, knowledge of harm caused by SHS exposure and attitudes towards tobacco control. A smoker was defined as “has smoked over 100 cigarettes and smoked in the last month”. Non-smokers included never smokers and former smokers.

### Related terms and measurements

Passive smoker refers to a non-smoker who inhaled the smoke exhaled from smokers on at least 1 day per week. The question in the questionnaire was “Typically, how many days per week did a smoker smoke in front of you? (1 day means in that day there were smokers smoking totally up to 15 min in front of respondents)”. Those who answered “1 day” or “more than 1 day” were regarded as passive smokers.

Household passive smokers were classified as passive smokers who were exposed often or sometimes to SHS in their household. The question in the questionnaire was “Typically, how often would a smoker smoke in front of you at home per week?”. Those who answered “Sometimes” or “Often” were regarded as household passive smokers.

Household SHS exposure rate was defined as the proportion of household passive smokers in the non-smoker population.

Knowledge of SHS exposure harm was assessed with the questions:

- “Do you think that children living with smokers are more likely to have asthma or other respiratory diseases?”
- “Do you think that women with a smoking husband are more likely to get lung cancer than other women?”
- “Do you think that passive smokers are more likely to have heart disease?”

Those who answered “yes” to all three questions were considered to have a good understanding of the harms of SHS exposure.

To study the respondents’ attitudes towards tobacco control policies, we asked three questions:

- “Do you agree with banning smoking in public places (ie, hospitals, schools, movie theatres, etc.) to protect the health of non-smokers?”
- “Do you agree with banning the selling of cigarettes to minors (<18 years old)?”
- “Do you agree with banning all cigarette advertisements?”

Those who agreed with all three policies were regarded as having a positive attitude towards tobacco control.

To measure household restrictions on smoking, we asked: “Which of the following best describes your household smoking restriction situations?” Those who answered “smoking not allowed in any indoor areas” were considered to have a smoke-free home. Those who answered “smoking allowed in some indoor areas” were considered to have a restricted home. Those who answered “smoking allowed in all indoor areas” were considered to have an unrestricted home.

### Data analysis

#### Descriptive analysis

We described the prevalence of household SHS exposure according to different demographic characteristics: provinces, gender, age, education level, occupation, marital status, urban or rural areas, and analysed the results using univariate analysis. The age and sex distribution in our survey differed from the actual situation, so we adjusted our samples using the national population data for 2000. Our survey sampled a 1:1 urban to rural ratio. This ratio did not represent the actual ratio, so we adjusted it to a 1:4 urban to rural ratio to reflect the national population data for 2000.

#### Analysis on the correlated factors of household SHS exposure

Dependent variables: we classified the status of the household SHS exposure as a dependent variable, 1 represented exposure, 0 represented non-exposure. Independent variables: we classified the possible correlated factors of household SHS exposure as independent variables, which were analysed using the logistic stepwise regression model. The entering and removing significance levels were 0.05. Dummy variables were set for multi-categories variables. The following three categories were introduced to our model: demographic characteristics (age, gender, marital status, place of residence (rural/urban), occupation, education level, province), knowledge factors (whether participants had received information about tobacco control, whether they knew that SHS exposure is harmful to the health, whether they had a strong understanding about the harm caused by SHS exposure) and attitude factors (whether participants had a positive attitude towards tobacco control, whether they agreed that doctors and teachers should act as role models for tobacco control, whether household smoking was completely prohibited).

## RESULTS

### Household second-hand smoke exposure by demographic characteristics

Among the 8142 non-smokers, 48.3% were exposed often or sometimes to SHS in their household. Further univariate analysis based on different demographic characteristics revealed that residence, gender, age, education, occupation and marital status were all related to household SHS exposure. Rural residents, females and married respondents reported high household SHS exposure. Higher education level showed a lower household SHS exposure rate. As for different occupations, government officials had the lowest reported exposure while farmers had the highest (table 1).

**Table 1:** Household second-hand smoke (SHS) exposure rate of people with different demographic characteristics

Demographic characteristics	Percentage of total (no. of subjects)	Weighted household SHS exposure rate (%)	χ^2^ Test	p Value
Residence:				
Rural	46.4% (3781)	49.3	10.0	0.002
Urban	53.6% (4361)	45.0		
Province:				
Sichuan	31.8% (2586)	48.5	261.8	<0.001
Jiangxi	32.9% (2678)	60.5		
Henan	35.3% (2878)	38.9		
Gender:				
Male	29.0% (2358)	42.9	48.8	<0.001
Female	71.0% (5784)	51.1		
Age:				
18–29	11.5% (936)	50.7	39.5	<0.001
30–39	29.9% (2438)	49.9		
40–49	24.0% (1953)	49.8		
50–59	21.5% (1752)	46.2		
60–69	13.1% (1063)	35.6		
Literacy:				
Illiterate or semi-illiterate	12.4% (1007)	51.6	11.4	<0.001
Primary	27.4% (2230)	51.4		
Junior High	32.9% (2675)	45.8		
Senior High	15.9% (1293)	48.7		
Junior College or above	11.4% (930)	45.5		
Occupation:				
Doctors/teachers	5.6% (457)	46.9	13.8	0.003
Government officials and state institution staff	6.9% (558)	38.7		
Farmers	43.5% (3537)	49.6		
Others	44.0% (3578)	47.3		
Marital status:				
Married	90.0% (7311)	49.8	113.4	<0.001
Divorced/widowed	5.5% (448)	19.8		
Single	4.5% (362)	49.1		
Total	8142	48.3		

Unknown or missing observations were not taken into the analysis in table 1. Therefore, the total of subjects in every demographic characteristic might not exactly equal 8142 people.

### Respondents’ knowledge of the harm caused by SHS exposure and attitudes towards tobacco control policies

A total of 57.8% of non-smokers knew that children living with smokers would be more likely to have asthma or other respiratory diseases; 44.9% knew that women with a husband who smoked would be more likely to get lung cancer than other women; 27.5% knew that passive smokers would be more likely to have heart disease. Only 21.9% of the respondents answered all three questions correctly, and 84.4% of respondents supported all three policies (banning smoking in public places, banning the selling of cigarettes to minors, banning all cigarette advertisements).

### Behaviours related to household SHS exposure

Of the 8142 non-smokers, 3165 said they had smoking family members. Among these 3165 respondents, 87.2% reported that smokers smoked in front of them. Of these 3165 respondents, 2124 had children in their home and 76.5% of these respondents reported that smokers smoked in front of their children. A total of 42.1% of non-smokers said that they would offer cigarettes to their guests. Among those with family members who smoked, 74.0% said they would ask the smokers not to smoke in front of them, 46.8% would ask smokers to smoke outdoors and 72.8% would try to persuade smokers to quit smoking (table 2).

**Table 2:** Non-smokers and smoking behaviour in the family

	No. of subjects	Often (%)	Sometimes (%)	Never (%)
Non-smokers offered cigarettes to guests	8099*	13.1	29.0	57.9
Non-smokers asked smokers not to smoke in front of them	2966†	37.1	36.9	26.0
Non-smokers suggested smoker smoke outdoors	2955†	17.5	29.3	53.2
Non-smokers tried to persuaded smokers to quit smoking	3054†	36.2	36.6	27.2

*The number of subjects was not exactly 8142 because of unknown and missing subjects; †the number of subjects was not exactly 3165 because of unknown and missing subjects.

### The relationship between household smoking restrictions and SHS exposure

Our survey found that only 6.3% respondents completely prohibited household smoking. Further analysis of the relationship between household smoking restriction and SHS exposure showed that the household SHS exposure rates in the case of “smoking allowed in SOME indoor places” and “smoking allowed in ALL indoor places” were higher than in the case of “smoking not allowed in ANY indoor areas” (table 3).

**Table 3:** The relationship between household smoking restrictions and second-hand smoke (SHS) exposure

Household smoking restriction	Household smoking restriction, % (no. of subjects)	Household SHS rate (%)	χ^2^ Test	p Value
Smoking not allowed in ANY indoor areas	6.3% (496)	20.0	93.0	<0.001
Smoking allowed in SOME indoor places	16.9% (1321)	50.5		
Smoking allowed in ALL indoor places	76.8 (6022)	51.4		

The number of subjects was not exactly 8142 because of unknown and missing subjects.

### Analysis of factors correlated with household second-hand smoke exposure

Multivariate analysis showed that nine factors were statistically significant: province, gender, age, marital status, education level, occupation, SHS exposure health risk knowledge, tobacco control policies advocacy status and household smoking restriction status. Females were more likely to be exposed to household SHS than males (odds ratio (OR) 2.04, 95% CI 1.8 to 2.23). The elderly population were less exposed to household SHS (OR 0.79, 95% CI 0.67 to 0.91). When compared to unrestricted houses, complete smoking restriction was a protective factor to help reduce household SHS exposure (OR 0.18, 95% CI 0.14 to 0.24). Better knowledge, attitudes and household restriction were associated with more reporting of SHS exposure. Table 4 shows the detailed results.

**Table 4:** Results of multivariate non-conditional stepwise logistic regression analysis

Correlated factors	Regression coefficient	SEM	Odds ratio (95% CI)	p Value
Demographic factors:				
Region				
Henan Province			1.00 (reference)	
Sichuan Province	0.35	0.06	1.43 (1.27 to 1.61)	<0.001
Jiangxi Province	0.71	0.06	2.04 (1.82 to 2.30)	<0.001
Gender				
Male			1.00 (reference)	
Female	0.69	0.05	2.00 (1.80 to 2.23)	<0.001
Age				
18–29 years old			1.00 (reference)	
60–69 years old	−0.24	0.08	0.79 (0.67 to 0.91)	<0.01
Literacy				
Illiterate or semi-illiterate			1.00 (reference)	
High school and above	−0.12	0.05	0.89 (0.80 to 0.98)	<0.05
Marital status				
Married			1.00 (reference)	
Divorced, widowed	−1.19	0.13	0.30 (0.24 to 0.39)	<0.001
Occupation				
Doctors/teachers			1.00 (reference)	
Farmers = 1	0.42	0.06	1.52 (1.34 to 1.72)	<0.001
Others = 1	0.18	0.07	1.91 (1.05 to 1.36)	<0.01
Respondents’ knowledge:				
Did not correctly answer all the three questions about the knowledge of the harm caused by SHS exposure			1.00 (reference)	
Correctly answered all the three questions about the knowledge of the harm caused by SHS exposure	0.13	0.06	1.14 (1.02 to 1.28)	<0.05
Respondents’ attitudes towards tobacco control:				
Policy attitude				
Did not support all three tobacco control polices			1.00 (reference)	
Supported all the three tobacco control polices	0.21	0.07	1.24 (1.08 to 1.42)	<0.01
Household smoking restriction				
Allow indoor smoking in some or all indoor places			1.00 (reference)	
Completely forbid indoor smoking	−1.71	0.14	0.18 (0.14 to 0.24)	<0.001

SEM, standard error of the mean.

## DISCUSSION

Our study showed that among the 8142 non-smokers, 48.3% were exposed often or sometimes to SHS in their household. Currently, national monitoring data on SHS exposure are quite limited. Although there are many studies on SHS exposure in developed countries, it is difficult to compare their findings as they often use different methods and definitions for household SHS exposure. However, based on the literature, we did find that these six counties had a much higher household SHS exposure level than in developed countries such as the US and New Zealand,^10^^–^14 and even higher than in some developing countries or regions such as Mexico.^15^ ^16^ Our survey also revealed a slightly higher household SHS exposure rate than reported in the 1996 and 2002 nationwide surveys.^5^ ^6^

People’s knowledge about the harm of SHS and their attitudes toward tobacco control are fundamental factors in reducing SHS exposure. Consistent with previous studies,^5^ ^6^ our survey showed that respondents’ knowledge about the harm of SHS was very limited. Only 21.9% of the respondents answered all three health risk knowledge questions correctly. Therefore all types of media should be used to publicise and educate about SHS exposure, warning people about the harms of smoking and SHS exposure, raising people’s level of awareness about avoiding SHS exposure, with the aim of reducing SHS exposure. By contrast, respondents had a positive attitude towards tobacco control policies; 84.4% supported all three tobacco control policies, which, suggests a strong basis for the establishment of tobacco control policies.

Smokers’ and non-smokers’ behaviours influenced household SHS exposure level. Similar reports on this aspect are absent in China. Our survey revealed that non-smokers reported high rates of smokers smoking in front of them or children. This was much higher than reported for other countries.^17^ ^18^ The rate of non-smokers asking smokers to smoke outdoors was low.

As for smokers, quitting smoking usually requires help since smoking is addictive. Many of the county areas of China do not have the counselling, medication and information resources to tackle nicotine addiction. As a result, it is difficult for smokers to quit smoking. However, they could be told not to smoke in front of others, which would reduce household SHS exposure. SHS does not have a “safe exposure” level. Nor can it be eliminated through ventilation and/or filtration. The health of non-smokers can only be protected through completely smoke-free environments.^19^ Therefore, in order to reduce household SHS exposure, every non-smoker needs to feel empowered to dissuade smokers from smoking indoors, telling them to smoke outdoors.

In many regions of China, offering cigarettes to guests is a common social custom. Offering and receiving cigarettes are regarded as polite behaviours and necessary forms of social interaction. According to our findings, 42.1% of non-smokers would offer cigarettes to guests. In other countries, it is extremely rare that a non-smoker would offer cigarettes to smokers. This revealed that smoking was not regarded as a bad habit in the selected six counties. We need to enhance our education in these areas in order to change this social norm.

In our survey, only 6.3% respondents completely forbade smoking indoors, which was similar to the rate in other developing countries but much lower than in developed countries.^20^^–^22 Many studies have demonstrated that restricting smoking at home is an effective way to reduce household SHS exposure. Furthermore, it could also help to reduce smoking experimentation by children, and to encourage smokers to quit smoking. The number of tobacco-free families are increasing in some developed countries.^23^^–^25

The findings of the analysis of the demographic factors and household exposure (including provinces, gender, age, marital status, education level and occupation), were consistent with previous studies.^5^ ^12^ ^26^^–^28 Henan Province had the lowest household SHS exposure level. This might be because the selected counties in the Henan Province (Xinan and Yanshi), were demonstration regions in the World Bank’s Project 7 “Disease Prevention Project” which included tobacco control activities. In 2000, the smoking rate in these counties was 26.08%.^29^

Females had a significantly higher household SHS exposure level than males. This phenomenon could be explained by the fact that in the county area of China most women are housewives and thus spend most of their time at home. As for age, elderly people usually had a lower household ETS exposure than young people.

**What this paper adds**

- The high prevalence of passive smoking and low level of knowledge in China is already known.
- The paper places focus on household second-hand smoke exposure, the new findings of this paper are the common practice of offering cigarettes, and the lack of action against smoking in the home. Additionally, this paper considers its correlated factors.

Analysis of the different occupations showed that farmers had the highest household SHS exposure, while doctors and teachers, who are supposed to be role models for tobacco control, had a surprisingly high household SHS exposure rate (46.9%), though this rate was less than for other occupations. Tobacco control experiences from developed countries suggested that the reduction of doctors’ smoking rates was a necessary precursor to reducing the entire population’s smoking rate.^30^^–^32 Doctors and teachers play a special role in society, and so it is important for them to set an example for others through their behaviours. Based on our results, we recommend that women, doctors and teachers should be our target groups for tobacco control interventions.

Somewhat unexpectedly, those who answered correctly all three questions about the harm caused by SHS exposure and those who supported all three tobacco control policies had high household SHS exposure. This phenomenon could be due to two reasons. First, respondents’ knowledge and attitudes towards tobacco control was not sufficient to affect the way they avoided SHS exposure. Currently in China, although some people understand the hazards of SHS exposure and have positive attitudes toward tobacco control, they still lack active and determined actions against SHS exposure. Second, this phenomenon might have been caused by our self-report survey method. The more knowledge one had about SHS exposure, the more likely a respondent might report such exposure.

A survey conducted by Shelley in 2003 on household smoking restrictions in Chinese-American families demonstrated that the household SHS exposure in tobacco-free households (7%) was significantly lower than for those that allowed smoking in some (68%) or all(73%) indoor places.^33^ Our survey showed similar results, suggesting that a completely smoke-free household policy is necessary in order to reduce household SHS exposure.

Our survey was a cross-sectional research study. Investigators conducted face-to-face interviews to collect information using a standardised questionnaire. Several limitations in this study need to be considered. First, although interviewers were well-trained and instructed in the same way, each interviewer may have had a different understanding of the survey questions which could result in bias in collection of the information. Second, our survey relied on self-report by respondents. Knowledge and awareness of respondents may have led to differences between perceived and actual SHS exposure levels level. Third, household SHS exposure was correlated with a number of factors. Even though factors such as age, sex, region and education level were controlled, there may have been some unknown factors that resulted in potential biases. Despite these limitations, our study provides scientific evidence for the need to develop an intervention plan for reducing household SHS exposure in county areas of China.
